# Assessment of the Predictive Potential of Pediatric Relative Fat Mass Compared to Alternative Measures of Obesity for Cardiorespiratory Fitness in Children: Longitudinal Associations During Two-Year Follow-Up

**DOI:** 10.3390/nu18050857

**Published:** 2026-03-06

**Authors:** Maria Zadarko-Domaradzka, Marek Sobolewski, Emilian Zadarko

**Affiliations:** 1 Faculty of Physical Culture Sciences, Medical College, University of Rzeszów, 35-959 Rzeszów, Poland; 2 Department of Quantitative Methods Rzeszów, University of Technology, 35-959 Rzeszów, Poland; msobolew@prz.edu.pl

**Keywords:** obesity, CRF, RFMp, health promotion, anthropometric measurements

## Abstract

Background/Objectives: Relative Fat Mass (RFM) is an anthropometric index estimating whole-body fat percentage. Though RFM is analyzed in scientific articles in various contexts, the research on the association between RFM and cardiorespiratory fitness (CRF) level is extremely limited. The aim of this study was to investigate the prognostic value of the relative fat mass pediatric index (RFMp) in predicting CRF results over a two-year period among school-age children, in comparison with alternative indices. Methods: Based on data comprising student measurements collected previously, in the years 2017–2019, a multiple regression analysis was conducted. Predictive models for CRF were constructed over a two-year period, separately for each of the eight indicators associated with obesity assessment. The models were prepared for boys and for girls separately. Results: over 40% of girls and boys have a BMI above the norm. In the case of both girls and boys, RFMp turned out to be the best CRF predictor over a two-year period. It proved to be better in terms of its predictive power than body mass index (BMI), body fat percentage (%BF), waist circumference (WC), waist-to-height ratio (WHtR), waist-to-hip ratio (WHR), tri-ponderal mass index (TMI) and waist-BMI ratio. Conclusions: RFMp demonstrated a certain advantage in terms of predictive ability compared to alternative indicators. This indicates its potential for implementation in the general pediatric population and clinical practice for the prediction of CRF. However, this needs to be confirmed in further studies involving a larger and more diverse population.

## 1. Introduction

Unhealthy eating habits combined with low physical activity (PA) in children increase the risk of obesity, diabetes, cardiovascular disease, and other conditions [1]. Data on the prevalence of obesity among children and adolescents in the European region are alarming. Between 2022 and 2024, one in four (25%) children aged 7–9 years were living with overweight (including obesity). Results by sex showed a higher prevalence of overweight among boys (26%, overall estimate) compared to girls (23%) [2]. In Poland, children aged 7–9 show an increase in excess weight with age (from 27.6% in 7-year-olds to 34.8% in 9-year-olds) and a decrease in PA participation. Among 9-year-olds, only 19.8% spend time on moderate physical activity 7 days a week [3]. An important role in the development of obesity in children is played by an insufficient consumption of vegetables and fruit, eating too many high-calorie snacks, an insufficient number of meals, skipping breakfast, drinking sugar-sweetened beverages, eating in front of the TV, and leading a sedentary lifestyle [4,5]. Results from some studies suggest that children with higher body fat do not consume more energy from food and drinks than their slimmer peers, but are less active [6]. It is increasingly emphasized that overweight and obesity are primarily a reflection of social and commercial determinants of health [7,8]. The excess of accumulated fat tissue causes a number of metabolic disorders and can lead to the development of a range of diseases [5].

Childhood obesity increases the incidence of cardiovascular disease risk factors and morbidity and mortality in adulthood [9]. The combination of overweight and physical unfitness in children aged 7–10 years increases the incidence of cardiometabolic risk factors, such as altered HDL-c and triglyceride levels (TG) [10]. Excess body fat has been shown to be negatively associated with cardiorespiratory fitness (CRF) in preschoolers, school-age children, and adolescents [11,12,13]. The importance of CRF is widely described in research and has been considered the basic index in evaluating health condition of youth, defined as ‘clinical vital signs’ [14,15]. Regular measurement of CRF as a marker of health and routine prescription of PA could be a prudent strategy to support public health. However, CRF is not routinely measured in healthcare settings [16]. Both body fat and CRF are key factors influencing cardiovascular health in childhood [17]. A good CRF level may reduce the incidence of cardiometabolic disorders in overweight children [10]. CRF levels in the adolescent and child population are not only indicators of one’s current health condition but they can also help in predicting future diseases [18].

Most assessments of obesity in terms of health are based on obesity defined by body mass index (BMI). The accuracy of BMI as a measure of body fat is a matter of debate [19,20]. It is most often used as a proxy for body fat in epidemiological studies, but it provides insufficient information about health at the individual level [21]. There is a need to better define obesity than using BMI alone [21,22,23,24,25]. BMI alone is an insufficient diagnostic criterion, and the distribution of body fat has a significant impact on health [24]. An alternative to BMI may be Relative Fat Mass (RFM) [26].

RFM is a relatively recently developed algorithm based on the measurements of body height and waist circumference, suggested as a new method of estimating body fat [27]. The authors developed several linear equations tailored to specific age groups (for both sexes combined and separately for females and males), namely: adults and adolescents aged 15–19, and the pediatric population aged 8–14, defining the algorithm for the latter as Relative Fat Mass pediatric (RFMp) [28]. The researchers demonstrated the existence of a good correlation between fat mass percentage (%FM) measured by means of dual-energy X-ray absorptiometry (DXA) and fat mass percentage estimated with RFM and RFMp among people of varied ethnic backgrounds [27,28]. Their findings were confirmed by an external RFM validation among a Mexican adult population, indicating a stronger correlation of RFM with DXA than with Air-Displacement Plethysmography (ADP) or with Bioelectrical Impedance Analysis (BIA) [29]. The results of studies among Brazilian children and adolescents have also confirmed that the equation developed for the pediatric population of both sexes, i.e., RFMp, is accurate in predicting %BF when compared to DXA at various stages of biological maturation [30].

In other prospective studies involving 531 children from a public school in Mexico, the highest correlation was found between BIA and RFM. The authors suggest that in the absence of BIA, RFM/pRFM can be used to obtain a better approximation of the percentage of fat mass [31]. According to Cho et al. [32], RFM reflects abdominal obesity and can be used in longitudinal/interventional studies involving adolescents, eliminating the need for costly imaging tests.

RFM can be used as a simple and intuitive marker of obesity and cardiovascular risk in the general population [33].

The analysis of the relationship between RFM and all-cause mortality in the general population of the Netherlands showed that higher RFM values are significantly associated with an increased risk of death [34]. Further analyses of another population have confirmed that correlation, showing that RFM outperforms conventional obesity metrics in estimating mortality risk related to, among others, diabetes and cardiovascular disorders [35].

Though RFM is analyzed in different contexts across studies [36,37,38,39,40,41,42], there are few studies on the association of RFM with the level of cardiorespiratory fitness (CRF) in both the adult and the pediatric population. The review of the available literature shows that until now no research has been conducted on analysing RFMp’s potential as a cardiorespiratory fitness predictor among school-age children in a longitudinal study. Our previous analyses, based on cross-sectional studies, revealed that among school-age children, the RFMp equation as juxtaposed with alternative anthropometric indicators of obesity, namely body mass index (BMI), waist-to-hip ratio (WHR), waist-to-height ratio (WHtR), tri-ponderal mass index (TMI), and waist-BMI ratio turned out to be the strongest CRF predictor, comparable with body fat percentage (%BF), obtained using the BIA method [43].

The aim of this study was to examine the prognostic value of RFMp for predicting CRF over a two-year period in school-age children in comparison with alternative indices.

## 2. Materials and Methods

### 2.1. Subject

The research was carried out in the south-eastern part of Poland. The analysis comprised the results of a longitudinal study covering children of school age participating in obligatory physical education classes. The sample selection was purposeful, and the research was conducted in a school with which the Faculty of Physical Education of the University of Rzeszów concluded a cooperation agreement. The study was conducted in the years 2017–2019, having obtained the approval of the university’s bioethics committee (approval number 1/06/2014). Prior to the tests, written consent of the school authorities, of the parents or legal guardians, and of the children was obtained.

Three rounds of measurements were conducted at one-year intervals (2017, 2018, 2019), always during the same months. Each time anthropometric measurements were performed, together with a cardiorespiratory fitness test assessed on the basis of the 20-m Shuttle Run Test (20mSRT). The main index of the cardiorespiratory fitness was the number of laps of the 20mSRT run (completed).

The inclusion criteria for the particular results to be analyzed were the calendar age of the children (during the first study they were aged 9–13, due to the RFMp algorithm being dedicated to children and adolescents aged 8–14) and the participation in all three measurements in consecutive years of the research. The analysed data concerned the same group of children. Incomplete data constituted an exclusion criterion. In total, 107 children (44 boys and 63 girls) met the inclusion criteria (Table 1).

### 2.2. Procedure

The research procedure, as well as the technique of the anthropometric measurements and the measurement of CRF, have been thoroughly described elsewhere [44].

In brief, the measurements were performed in three consecutive years by the same trained staff, according to a standardized protocol.

Body height (BH) was measured with a mobile stadiometer (SECA 213, Hamburg, Germany) with accuracy to 0.1 cm, and body weight (BW) and body fat percentage (%BF) were determined by means of the bioelectrical impedance analysis (BIA) method with the use of a body composition analyzer, Tanita TBF 300 (Tanita Corporation, Tokyo, Japan). Waist and hip circumferences (WC and HC) were measured using the Gulick anthropometric tape (BASELINE) with accuracy to 0.1 cm.

CRF was assessed on the basis of the 20mSRT [45]. The participants of the test performed a shuttle run over the distance of 20 meters, established with a measuring tape and marked with a line and cones. The speed of the run was regulated by means of audio signals, which occurred increasingly frequently. The starting speed of the run was 8.5 km/h, which was increased by 0.5 km/h with each stage. The result of the test consisted in the number of fully completed 20-m laps.

BMI was used to assess the nutritional status of the children studied. BMI z-scores were calculated and classified according to World Health Organization (WHO) standards.

### 2.3. Statistical Analysis

Baseline data on age, somatic measurements, and body fat percentage from the first examination were presented using means and standard deviations, and the significance of differences between boys and girls was assessed using the Mann–Whitney test. The number of laps measured two years later was presented in the same manner. The significance of changes in BMI classification over a two-year period was assessed using the Wilcoxon test.

In order to determine the indices that describe the results of the CRF test over a two-year period, linear regression analysis was applied. The models were prepared for boys and girls separately. The dependent variable was the number of completed stages after a two-year follow-up, while the independent variables were age, as a covariate that has a very clear impact on the performance test result, and separately each of the somatic indicators (RFMp, BMI, WHR, WHtR, TMI, Waist-BMI Ratio) as well as %BF in the first study.

Based on the data obtained through anthropometric measurements, indices related to obesity assessment were calculated according to the following formulas:

RFMp = for girls and boys: 74 − (22 × (height/waist)) + (5 × sex)

where sex equals 0 for boys and 1 for girls

BMI = body weight (kg)/body height^2^ (m)

WHR = waist circumference (cm)/hip circumference (cm)

WHtR = waist circumference (cm)/body height (cm)

TMI = weight (kg)/height^3^ (m)

Waist-BMI Ratio = waist circumference (cm)/body mass index (kg/m^2^)

An attempt was also made to find a forecasting model in which a larger number of indicators would occur simultaneously. For this aim, a stepwise regression procedure (both backward and forward) was used to identify the model that best explained the dependent variable while including only statistically significant independent factors. The predictive value of particular indicators was assessed based on the coefficient of determination (*R*^2^). All analyses were performed in the Statistica 13.3 software (TIBCO Software Inc., Palo Alto, CA, USA). The threshold for statistical significance was set at α = 0.05.

## 3. Results

### 3.1. General Somatic Characteristics of the Group

Based on the data obtained from the anthropometric measurements at the beginning of the study (BH, BW, WC, HC and BIA), indices related to obesity assessment were calculated (Table 2). Statistically significant differences between boys and girls for some somatic indicators, as well as body fat content, confirm the need to model the CRF test results separately for each gender.

According to the BMI classification, the largest percentage in the study group were children with normal body weight, among both boys (54.5% at baseline and 59.1% after two years) and girls (57.1% at baseline and 65.1% after two years). However, the percentage of overweight or obese children was also high (boys 45.5% vs. girls 42.9 at baseline and 40.9% vs. 34.9% after two years), with a relatively higher incidence of obesity among boys. No below-normal values were recorded in the entire group. During the two-year observation period, the distribution of BMI categories among girls changed significantly (*p* = 0.015)—the proportion of girls with a normal BMI increased, while the proportion of overweight and obese girls decreased. Among boys, the percentage of obese individuals remained unchanged (Table 3).

The result of the test CRF consisted in the number of fully completed 20-m laps. Table 4 presents the mean value, standard deviation, and the number of run (completed) laps of the 20mSRT in each age group among the girls and the boys after a two-year follow-up period. The number of completed laps shows considerable variation with respect to the children’s age. With increasing age, the dominance of boys is also becoming apparent.

### 3.2. Predictive Models

First, eight predictive models for the CRF over a two-year period were constructed, separately for each of the analyzed somatic indicators. The models were prepared for boys and girls separately, obviously introducing additionally the children’s age as a covariate (Table 5).

All the predictive models considering the analyzed anthropometric indices were statistically significant; however, for both the girls and the boys, RFMp seems to be the best CRF predictor over the two-year period; with the accuracy of the prediction of the future value of the laps considerably higher for the boys: coefficient of determination at the level of *R*^2^ = 71% vs. *R*^2^ = 30.7% for the girls.

In order to more precisely identify the factors that best predict CRF test results over a two-year period, multivariate regression analysis was applied. The models were prepared separately for girls and for boys. The dependent variable was the number of laps two years after the measurement of anthropometric indicators, and the independent variables: age and values of all somatic and body build indices at the beginning of the study. The stepwise regression procedure was employed to determine the model best explaining the dependent variable, while incorporating statistically significant independent variables. However, no more complex models than those presented in Table 4 were found. As such, detailed results for the models with the highest coefficient of determination, based on age and RFMp, are presented below.

The variant obtained for the boys is capable of providing very good descriptions of CRF over a period of two years. The coefficient of determination equaled *R*^2^ = 71.5%, which constitutes a very strong result. The model also comprised age (which seems quite natural, as together with physical development the raw CRF test results are higher and higher) and RFMp. The increase in RFMp by 1 point transfers to the decrease in the number of completed laps by 2.9 (Table 6). Based on the value of the *ß* coefficient, it can be concluded that in the group of boys, the initial RFMp value (*ß* = −0.72) had a significantly greater impact on the CRF result after two years than age (*ß* = 0.22). 

The prognostic value of the model obtained for girls is much lower, with the coefficient of determination at the level of *R*^2^ = 30.7%. The model comprised age, as well as RFMp. The increase in RFMp by 1 point transfers to the decrease in the number of completed laps by 0.8 (Table 7). In the group of girls, the effect of RFMp on CRF after two years was slightly greater than that of age (*ß* = −0.43 and 0.30), although this difference was not as pronounced as among boys.

## 4. Discussion

In this study, we sought to estimate the predictive value of RFMp in predicting CRF over a two-year period in school-aged children compared to alternative indicators, and to determine whether there were gender differences within the study group. A significant advantage of our study is that it is the first to show the relationship between RFMp and CRF expressed in children in terms of the number of 20m laps fully completed two years in advance.

In this study, all the predictive models including the examined anthropometric indices in the context of estimating CRF were statistically significant. It is worth noting that in all the models, the accuracy of estimates for the future Laps value (the number of completed laps of 20mSRT) is considerably higher for boys than for girls. We have demonstrated that from among all the obesity measures considered, the RFMp equation and the WHtR showed the highest predictive value for CRF over a two-year period (respectively: boys: *R*^2^ = 71.5%; girls: *R*^2^ = 30.7% and boys: *R*^2^ = 68.7%; girls: *R*^2^ = 30.4%).

Similar studies, but in a cross-sectional study of Welsh children aged 10–11, taking into account only indicators such as BMI, WC, WHtR, and %BF, showed that the strongest predictors of CRF levels were, in order, BMI (*R*^2^ = 69.1%), BF% (*R*^2^ = 65.3%), WHtR (*R*^2^ = 61%), and WC (*R*^2^ = 60.1%), emphasizing that they were all statistically significant [46].

Low physical fitness in children is associated with the development of cardiometabolic risk factors and that risk can be modified through the improvement of CRF [47]. It has been observed that CRF functions as a significant partial mediator in the association between obesity and cardiometabolic risk among European adolescents [48]. In a three-year observation, it was noticed that CRF moderates the association between %BF and the systolic and diastolic blood pressure among children and adolescents aged 8–17. Changes in CRF attenuated the association between baseline %BF and blood pressure during the observation period [49]. A study of a Chinese pediatric population aged 8–10 (N = 1557) has shown that CRF partially weakens the association between adiposity indices, i.e., BMI, body fat mass index (BFMI), or WHtR and individual and group outcomes of cardiometabolic risk markers (CMR), with WHtR having the most significant mediating effect [17].

Given direct measurements of fat tissue and CRF require specialist equipment and time, new measures of obesity, based on simple anthropometric measurements, are searched for and predictive models are employed, allowing for estimations of CRF based also on simple anthropometric measurements [50,51,52,53].

In studies of children and adolescents, the 20m-Shuttle Run Test is most commonly used for the assessment of CRF [17,53,54,55], and the results obtained so far support the application of this test as a holistic health indicator among children and adolescents [56]. However, in order to perform this test, 20 m of open space are needed, which constitutes a certain limitation, especially in clinical conditions. That is why there is a search for new methods of CRF estimation, based on easily accessible information that could be gathered during a GP appointment, such as age and BMI, rather than on fitness tests, in order to make use of this important biomarker in clinical and environmental practice [57].

From a methodological point of view, RFMp is a promising anthropometric proxy variable that can be used in longitudinal, interventional, and epidemiological studies, especially where direct measurement of CRF is difficult, costly, or burdensome for participants. Its measurement versatility (BH and WC are routinely recorded in most studies involving children) makes RFMp easy to integrate into various research protocols and datasets.

The outcomes of the longitudinal work presented in this study confirm our previous findings from cross-sectional studies. Previously constructed models, in which the number of laps constituted the dependent variable, while sex and age were the independent variables, RFMp turned out to be of the highest predictive value for CRF (*R*^2^ = 51.1%), followed by WHtR (*R*^2^ = 50.0%) [43]. Similarly to this longitudinal study, in the other one the prognostic value of the model obtained for boys was much higher than for girls with the coefficients of determination amounting to, respectively, *R*^2^ = 52.9% and *R*^2^ = 32.9% [43]. The epidemiology of heart disease shows that it affects men more frequently. Therefore, the fact that it is possible to predict, two years in advance, which boys are likely to perform worse on the CRF test appears particularly valuable. The results of a nationwide study conducted in 2022–2023 among Polish children aged 7–9 indicate that in all age groups, excess weight and elevated systolic blood pressure are more common in boys than in girls. Average waist and hip circumference values are also significantly higher among boys than girls in all age groups. In addition, boys are significantly less likely to eat fruit and vegetables [3].

Both measures that had the highest predictive value in our models, that is, RFMp and WHtR, are based on two simple somatic measurements: body height and waist circumference. In their studies, Wollcott and Bergman [28] noted that RFMp and WHtR demonstrated a similar ability to predict whole-body fat percentage in girls and boys. Among children and adolescents aged 8–14, RFMp had a better diagnostic accuracy for overweight or obesity for both sexes, as compared to BMI.

Additionally, studies among adults have shown that RFM, when compared to BMI, was a better %BF predictor, determined using various methods of body composition analysis [29]. It has been noted that, among numerous anthropometric indices associated with obesity, i.e., BMI, WHR, RFM, WC, BSI, and WWI, it is RFM that shows the strongest correlation with the risk of incident heart failure among a Dutch population [58]. RFM was a stronger indicator and predictor of cardiometabolic multimorbidity (CMM) risk than BMI [59].

Further findings indicate that RFM provides high predictive accuracy in the case of dyslipidaemia and metabolic syndrome in both sexes [60]. It is also strongly correlated with dyslipidaemia and glycemia in patients with type 2 diabetes [61]. RFM is strongly correlated with the risk of incident heart failure [58]. A cross-sectional study showed a significant positive correlation between RFM and stroke; therefore, considering RFM levels might help prevent the occurrence and recurrence of stroke [62].

Based on our own studies and the review of other researchers’ findings, it seems that the RFMp algorithm worked out by Woolcott and Bergman [28] is a tool that should be propagated and routinely used both in clinical and school practice in order to estimate fat tissue and predict CRF in the population of children to identify as early as possible people/environments requiring intervention. The simplicity of the measurements included in the RFMp algorithm allows it to be used in studies involving large, diverse, and multinational populations and enables a comparison of CRF predictions in the context of population differences in fat distribution, provided that appropriate external validation is performed. External validations are crucial to determine whether the algorithm maintains its predictive value in different somatic and epidemiological conditions. Furthermore, the adaptation of RFMp to other projects may lead to a more consistent and comparable assessment of surrogate indicators of obesity in population studies.

Our study shifts the discussion from the descriptive level (prevalence of overweight and obesity) to the prognostic level (the ability of the indicator to predict future CRF fitness), which may be important for the design of prospective studies and early risk identification strategies.

Early identification of low cardiorespiratory fitness and of excessive fat tissue in a pediatric population as well as the implementation of intervention measures increase the chance of preventing health problems in the future. Therefore, weight control and health promotion should start as early as possible, because excess body fat is negatively correlated with CRF [11].

School is the optimal place for simultaneous interventions concerning healthy eating habits, weight control, and CRF in order to develop health-related physical fitness (H-RF). Spreading knowledge about nutrition and healthy lifestyles and motivating children in Poland to change their attitudes and eating habits is one of the tasks aimed at preventing overweight and obesity under the National Health Program. The results of our research show that this problem is very real, as in the analyzed group, over 40% of girls and boys have a BMI above the norm. Although BMI is a commonly used indicator, it has limitations in assessing body fat and its distribution, which has a significant impact on health. This points to an urgent need to use other screening indicators. The classification of obesity based on BMI overlooks children and adolescents at increased cardiometabolic risk due to increased fat mass [63]. Our results indicate that the RFM algorithm, for example, may be an alternative to BMI in estimating CRF. RFMp can facilitate the early identification of children at risk of low CRF and thus an increased risk of cardiometabolic disorders. In a clinical setting, it can support decisions about referring children for further diagnosis or interventions aimed at improving physical health.

### Strong and Weak Point of the Study

To the best of our knowledge, our study is the first one that has shown whether the newly developed RFMp algorithm can predict the CRF in children over a two-year period, and has compared its predictive power with that of alternative anthropometric indices used to assess obesity. Providing the regression model coefficients makes it possible to use it in practice to estimate CRF. The ease of calculation, based solely on two anthropometric measurements, makes RFMp a potentially useful screening tool to support the early identification of children at risk of low CRF and metabolic complications. RFMp can serve as a practical anthropometric proxy variable in longitudinal and interventional studies investigating the relationship between obesity and physical fitness outcomes.

Despite promising results, the study has several significant limitations that may affect the interpretation and generalizability of the findings.

The small and ethnically homogeneous sample of children limits the external validity of the results. The lack of ethnic diversity is particularly important in the context of waist circumference-based indicators, as fat distribution and body proportions vary between populations. This may lead to an overestimation or underestimation of the predictive value of RFMp in groups with different anthropometric characteristics. The relatively small sample size may also have increased the risk of overfitting of the regression models. As a result, the predictive accuracy of the algorithm may be overestimated.

The lack of data on physical activity levels and biological maturity stage is a potential source of confounding error, as both factors are strong determinants of CRF. Their exclusion from predictive models may have led to an overestimation of the relationship between RFMp and CRF.

A two-year observation period, although valuable, may be insufficient to fully capture the dynamics of CRF and fat mass changes during a period of intensive growth. Longer observations would be more appropriate for assessing the predictive stability of RFMp.

In order to confirm the results obtained and unequivocally assess the scalability of the RFMp algorithm, studies on larger, more diverse populations and using more precise CRF assessment methods are necessary. Future studies should evaluate the ability of the RFM algorithm to predict CRF based on direct measurements of VO_2_max.

## 5. Conclusions

All the predictive models, including the examined anthropometric indices in the context of estimating CRF, were statistically significant. For both girls and boys of school age, RFMp proved to be the best CRF predictor over a two-year period. It demonstrated a certain advantage in terms of predictive power over BMI, %BF, WC, WHtR, WHR, TMI, and waist-BMI ratio, which indicates its potential for implementation in the general pediatric population and clinical practice for predicting CRF. However, this requires confirmation in other studies involving a larger and more diverse population

## Figures and Tables

**Table 1:** Number of children participating in all consecutive measurements.

Age (Years) at Baseline	Boys N (%)	Girls N (%)	Total N (%)
9	7 (15.9)	3 (4.8)	10 (9.4)
10	13 (29.5)	17 (27.0)	30 (28.0)
11	8 (18.2)	13 (20.6)	21 (19.6)
12	7 (15.9)	18 (28.6)	25 (23.4)
13	9 (20.5)	12 (19.0)	21 (19.6)
Total	44	63	107

**Table 2:** General characteristics of the group of boys and girls at the beginning of the study in 2017.

Features (At Baseline)	Sex		*p*
	Boys (N = 44)	Girls (N = 63)	
	M ± SD	M ± SD	
Age (years)	11.0 ± 1.4	11.3 ± 1.2	0.192
BH (cm)	148.2 ± 10.2	152.2 ± 8.8	0.021
BW (kg)	43.4 ± 11.2	46.2 ± 9.8	0.151
BMI (kg/m^2^)	19.6 ± 4.0	19.8 ± 3.3	0.471
BF (%)	15.8 ± 8.7	22.2 ± 7.7	<0.001
WC (cm)	66.4 ± 8.5	65.5 ± 7.8	0.535
WHR	0.83 ± 0.05	0.78 ± 0.05	<0.001
WHtR	0.45 ± 0.06	0.43 ± 0.05	0.199
TMI (kg/m^3^)	13.3 ± 2.7	13.1 ± 2.2	0.961
Waist-BMI (m/kg)	3.48 ± 0.27	3.34 ± 0.24	0.023
RFMp	24.5 ± 6.3	27.5 ± 5.9	0.011

Assessment of the significance of differences: *p*-value calculated using Mann–Whitney test, BH—body height, BW—body weight, %BF—percentage of body fat, BMI—body mass index, WC—waist circumference, WHR—waist-to-hip ratio, WHtR—waist-to-height ratio, TMI—tri-ponderal mass index, RFMp—relative fat mass pediatric, M—mean, SD—standard deviation.

**Table 3:** BMI percentile classification for boys and girls.

Classification BMI According to WHO z-Score	Boys (*p* = 0.123)				Girls (*p* = 0.015)			
	At Baseline		After Two Years		At Baseline		After Two Years	
	N	%	N	%	N	%	N	%
normal	24	54.5	26	59.1	36	57.1	41	65.1
overweight	9	20.5	7	15.9	17	27.0	14	22.2
obesity	11	25.0	11	25.0	10	15.9	8	12.7

Assessment of the significance of changes during two years: *p*-value calculated using Wilcoxon test.

**Table 4:** The mean (with standard deviation) of number of completed laps of the 20mSRT in each age group among the girls and the boys at two years of follow-up in 2019.

Age (Years)	Number of Laps				*p*
	Boys		Girls		
	N	M ± SD	N	M ± SD	
9	7	39.9 ± 18.4	3	41.0 ± 12.3	1.000
10	13	45.8 ± 19.8	17	32.7 ± 9.3	0.065
11	8	60.6 ± 15.8	13	35.8 ± 10.5	<0.001
12	7	74.9 ± 33.0	18	39.9 ± 9.9	0.041
13	9	70.7 ± 26.6	12	45.5 ± 14.3	0.028

Assessment of the significance of differences: *p*-value calculated using Mann–Whitney test Laps—laps of the 20mSRT, M—mean, SD—standard deviation.

**Table 5:** Predictive values of the CRF test (number of laps) with reference to particular somatic indicators measured two years earlier—results of regression analysis.

Model	Independent Factors	Statistics of Regression Models for Number of Laps After Two Years					
		Boys			Girls		
		*R* ^2^	*F*	*p*	*R* ^2^	*F*	*p*
1	Age, BMI	59.8%	30.4	<0.001	26.7%	10.6	<0.001
2	Age, %BF	54.4%	24.5	<0.001	22.2%	8.3	<0.001
3	Age, WC	38.1%	24.0	<0.001	21.7%	7.8	0.001
4	Age, WHR	59.3%	29.2	<0.001	18.5%	6.6	0.003
5	Age, WHtR	68.7%	43.9	<0.001	30.4%	12.7	<0.001
6	Age, TMI	59.0%	29.5	<0.001	28.6%	11.6	<0.001
7	Age, Waist-BMI Ratio	35.5%	11.5	<0.001	17.8%	6.3	0.003
8	Age, RFMp	71.5%	50.2	<0.001	30.7%	12.9	<0.001

*R*^2^—coefficient of determination, *F*, *p*—test statistic and *p*-value for significance of whole model, BMI—body mass index, %BF—percentage of body fat, WC—waist circumference, WHR—waist-to-hip ratio, WHtR—waist-to-height ratio, TMI—tri-ponderal mass index, RFMp—relative fat mass pediatric.

**Table 6:** Regression model for number of laps (boys).

Independent Factors (at Baseline)	Laps (Two Years Later) *R*^2^ = 71.5% *F* = 50.2 *p* <0.001		
	*B* (95% CI)	*p*	*ß*
Constant	83.856 (33.149; 134.563)	0.002	×
Age (years)	4.136 (0.567; 7.706)	0.024	0.22
RFMp	−2.903 (−3.678; −2.129)	<0.001	−0.72

*R*^2^—coefficient of determination, *F*—test statistic and *p*-value for significance of whole model, *B*—regression coefficient with 95% CI, *p*-value for significance of each regression coefficient, *ß—*value of the standardized coefficient, RFMp—relative fat mass pediatric.

**Table 7:** Regression model for number of laps (girls).

Independent Factors (At Baseline)	Laps (Two Years Later) *R*^2^ = 30.7% *F* = 12.9 *p* < 0.001		
	*B* (95% CI)	*p*	*ß*
Constant	29.102 (0.375; 57.828)	0.047	×
Age (years)	2.899 (0.747; 5.051)	0.009	0.30
RFMp	−0.847 (−1.287; −0.408)	<0.001	−0.43

*R*^2^—coefficient of determination, *F*—test statistic and *p-*value for significance of whole model, *B*—regression coefficient with 95% CI, *p*-value for significance of each regression coefficient, *ß—*value of the standardized coefficient, RFMp—relative fat mass pediatric.

## Data Availability

The data presented in this study are available upon request from the corresponding author due to ethical restrictions related to participant confidentiality. All data generated or analyzed during this study are included in this published article.

## Abbreviations

The following abbreviations are used in this manuscript:

RFM	relative fat mass
RFMp	relative fat mass pediatric
CRF	cardiorespiratory fitness
20mSRT	20-mShuttle Run Test
PA	Physical activity
BMI	body mass index
WHO	World Health Organization
WC	waist circumference
HC	hip circumference
WHtR	waist-to-height ratio
WHR	waist-to-hip ratio
TMI	tri-ponderal mass index
BH	body height
BW	body weight
BF	body fat
BIA	bioelectrical impedance analysis
DXA	dual-energy X-ray absorptiometry
ADP	Air-Displacement Plethysmography
CMR	cardiometabolic risk markers
TG	triglycerides
CMM	cardiometabolic multimorbidity
BSI	body-shape index
WWI	weight-adjusted-weight index
VO_2_max	maximal oxygen uptake
